# Electromagnetic Field Shielding Using Interior Paints Enhanced with Metal Powders

**DOI:** 10.3390/ma18163916

**Published:** 2025-08-21

**Authors:** Ján Zbojovský, Pavol Liptai

**Affiliations:** 1 Department of Electric Power Engineering, Faculty of Electrical Engineering and Informatics, Technical University of Košice, Letná 1/9, 042 00 Košice-Sever, Slovakia; jan.zbojovsky@tuke.sk; 2 Institute of Recycling and Environmental Technologies, Faculty of Materials, Metallurgy and Recycling, Technical University of Košice, Letná 1/9, 042 00 Košice-Sever, Slovakia

**Keywords:** electromagnetic shielding, absorption, reflection, metal powders, graphite-enhanced coatings

## Abstract

This article deals with the issue of electromagnetic radiation, specifically methods of eliminating radiation using protective coatings. Protective coatings were created from commercially available fabricated but also recycled metal powders and commonly available interior paint. The aim of the experiments was to produce protective coatings with different qualitative and quantitative compositions and subsequently test their shielding effects. For the preparation of the coatings, mixtures in the form of commercially produced powder with a particle size of <10 μm were used, namely aluminum oxide (Al_2_O_3_), manganese dioxide (MnO_2_), and graphite (C). Recycled powders are powdered iron (Fe) and zinc oxide (ZnO) with a particle size of <50 μm. The powders were mixed in various ratios and compounds into a commercially available white interior paint. Measurements were performed in the frequency range of 0.9–9 GHz with a step of 0.1 GHz, evaluating the shielding effectiveness, absorption, and reflection. The best shielding values were achieved for samples containing 100 g of carbon powder, 100 g of iron powder, and 100 g of manganese dioxide, ranging from 0.38 to 6.2 dB in the full measured frequency range.

## 1. Introduction

Electromagnetic radiation has long been a relatively unknown topic to the general public. However, in recent decades, the dramatic increase in wireless technologies such as mobile phones, Wi-Fi networks, and other communication systems has sparked increased interest in the subject, even outside professional circles. Mobile transmitters are often placed on residential buildings, which can raise concerns about the potential health risks associated with exposure to electromagnetic fields. Electromagnetic fields generated by different sources gradually interfere with each other. There is a problem called electromagnetic interference (EMI) [1]. This interference results in poor performance of electronic and electrical equipment. The widespread use of these technologies has pushed this electromagnetic pollution to levels never before achieved [2]. Several ways to protect against electromagnetic radiation are already applied in the design and production of electrical equipment. Manufacturers must comply with standards-setting limits for electromagnetic emissions, but completely eliminating radiation is not technically feasible. Moreover, the signal must not be entirely suppressed for the proper operation of mobile and wireless devices. Therefore, using shielding materials that can reduce the intensity of the electromagnetic field in a given environment appears to be an effective solution. Shielding can be achieved by weakening the electromagnetic waves transmitted from an electrical circuit either by reflecting the wave or by absorbing and scattering the radiation energy inside the material [3].

The issue of electromagnetic radiation shielding is extensively studied at a professional level in numerous scientific works. Numerous studies in the literature have been conducted on the issue of electromagnetic shielding and the development of materials that suppress this phenomenon. For example, Geetha et al. [1] provided a comprehensive review of methods and materials for EMI shielding, including polymer nanocomposites that combine low weight and high shielding effectiveness. Cilento et al. [4] investigated nanocomposites containing graphene nanosheets and their ability to absorb electromagnetic radiation in the X-band, with the highest absorption being achieved for disorganized structures. Jang et al. [5] demonstrated that metallic coatings, such as Cu-Zn, exhibit high shielding effectiveness, with the effectiveness increasing with the thickness of the coating. Zachariah et al. [6] analyzed hybrid materials for electromagnetic shielding, including polymers, cement, concrete, and paints, emphasizing their reflection and absorption mechanisms. A study by Bontas et al. [7] confirmed that graphene-modified epoxy coatings increase flexibility while providing excellent shielding effectiveness. Ölçen et al. [8] investigated electroplated coatings of Zn-Ni, Zn-Co, Ni-Co, and Zn-Ni-Co alloys, finding that Zn-Ni-Co coatings had the highest shielding effectiveness in the X-band, while Ni-Co coatings showed the best absorption properties. Sorgucu [9] investigates the improvement of electromagnetic interference (EMI) shielding effectiveness by coating aluminum oxide (Al_2_O_4_) with nanogold (AuNp). This study uses advanced analytical techniques such as SEM, EDX, and XRD to characterize the materials in detail. The results show that the coated Al_2_O_4_ has an increased shielding effectiveness of up to 12% compared to pure aluminum oxide, with absorption contributing more than reflection. An interesting study is the development of low-cost building materials with electromagnetic shielding and absorption in the X-band (8–12 GHz). The main goal was to use metallurgical slag and tire waste as additives in cement paste, thereby investigating their ability to reduce electromagnetic interference (EMI) [10]. The references provide an overview of other publications dealing with similar issues [11,12,13,14,15,16,17,18].

In general, it can be concluded that there is a limited number of studies addressing the development of interior paints for electromagnetic field shielding. This paper, therefore, focuses on investigating the effectiveness of experimental shielding coatings that could serve as an effective barrier against electromagnetic radiation indoors. The innovative potential of this study is also focused on the possibility of using recycled materials for electromagnetic shielding purposes, a topic that is currently under-researched. This argument is also supported by the analysis of studies in the introduction, which investigate the shielding effectiveness of various mixtures made only from commercially available materials from primary raw materials.

## 2. Materials and Methods

The selection of commercially available fabricated powder materials was made based on market availability and relatively low price. For the needs of this research, powders from various manufacturers were procured, namely MnO_2_, Al_2_O_3_, and graphite C with particle size < 10 μm and purity ≥ 99%. The specific parameters of MnO_2_ powder are D50 ~5 μm and D90 < 10 μm, and for Al_2_O_3_ powder, D50~1–1.8 μm and D90 < 10 μm. Recycled materials were selected from the perspective of easy availability and research focus at the home institute. For the purposes of this research, transformer sheets were used. The Fe powders were obtained by cutting into smaller pieces and then grinding on a planetary mill (Planetary Ball Mill PM 100, Retsch GmbH, Haan, Germany) and subsequently vibration sieving to a fraction size of <50 μm. ZnO was obtained by hydrometallurgical processing of hazardous waste originating from industrial activities, namely electric arc furnace dust and galvanizing flue dust. Detailed methods of obtaining ZnO by the hydrometallurgical method but with different procedures and analyses are described in previous research of the Institute of Recycling and Environmental Technologies, namely Pirošková et al., 2018 and 2022 [19,20], Oráč et al., 2021 [21], Kundráková et al., 2025 [22], and Vindt et al., 2017 [23]. Hydrometallurgical recycling of these industrial wastes obtains ZnO with a purity of 97–99%, as demonstrated by XRD and SEM-EDX analyses in these studies. The size of the fraction of the obtained ZnO product used in coatings was adjusted to <50 µm.

## 3. Shielding Effectiveness, Reflection, and Absorption of the Electromagnetic Field

The purpose of shielding is to prevent either complete or partial propagation of the electromagnetic field. The shielding effectiveness is defined as the ratio of the electric or magnetic field incident on the shielding to the electric or magnetic field transmitted through the barrier. Another way to determine the shielding value is to compare the magnitude of the electromagnetic radiation incident on the object under investigation without shielding to the magnitude of the electromagnetic radiation incident on the object under investigation with shielding. The primary purpose of shielding is to limit the radiation from the electronics of an electrical device to its surroundings through the device housing. This measure was introduced to eliminate emissions produced by the electrical device itself. Standards have been established to protect the population, and electromagnetic compatibility requirements have also been developed. Another goal is to protect sensitive electrical devices from external radiation through coupling, which could cause interference inside the device. To better clarify the issue, it is helpful to give an example. Sensitivity may be reduced, such as in radar or television transmitters. The electromagnetic shielding effect can be considered a linear system, where we can determine the so-called shielding coefficient *K*_S_. The shielding coefficient is the electric field intensity *E*_t_ ratio at a certain point to the field intensity *E*_i_ incident on the shielding barrier. This ratio also applies to the magnetic field [24,25,26].

*K*_s_ = *E*_t_/*E*_i_, or *K*_s_ = *H*_t_/*H*_i_ (1)

In practical measurements or calculations, the logarithmic measure of this coefficient is used. It is often referred to as shielding effectiveness.

*SE* = 20 *log*(*E*_i_/*E*_t_) [dB] (2)

Mathematically, it is possible to calculate the absorption coefficient *A*, reflection *R*, and shielding effectiveness *SE*. The general notation of shielding effectiveness can be calculated according to

*SE* = *A* + *R* [dB] (3)

where

*A*—is the absorption coefficient;
*R*—is the reflection coefficient.

## 4. Preparation of Experimental Samples and Setup of the Workplace

The procedure for preparing the experimental samples was as follows. First, it was necessary to mix the Primalex Plus interior paint in a 5 L container and dilute it according to the ratio indicated on the packaging as stated by the manufacturer. For each experimental sample, 400 mL of Primalex Plus paint was taken. Each powder was weighed according to the table below (Table 1), which shows the amount directly added to the measuring cup. The following scale was used for weighing: After adding the powder to the paint, mixing was first carried out using a VarioMAG MONO magnetic stirrer (H + P Labortechnik GmbH, Oberschleißheim, Germany). The mixture was then further homogenized by ultrasonic dispersion PS-10A (GT SONIC, Shenzhen, China). The PS-10A operates at a frequency of 40 kHz with a tank volume of 3 L, ensuring thorough dispersion of the powders in the liquid medium. The total mixing time was approximately 10 min per sample. The prepared coatings were applied to MDF boards (medium-density fiberboard) with dimensions of 100 cm × 100 cm and were dried at room temperature for 24 h before measurement. Each fiberboard was coated with 400 mL of the prepared coating mixture. After the individual samples were created, measurements were gradually carried out. The electromagnetic shielding measurements were performed in a COMTEST Model 1710-100 anechoic chamber, (COMTEST Engineering, Zoeterwoude, The Netherlands) which ensures minimal signal reflection. An Agilent N5183A MXG analog signal generator (Agilent Technologies, Santa Clara, CA, USA) with a frequency range set from 0.9 to 9 GHz and a step of 0.1 GHz was used as the source of electromagnetic waves. The radiated signal was transmitted through an RF Spin DRH18-E (RF SPIN s.r.o., Prague, Czech Republic) horn antenna (impedance 50 Ω, max. input 100 W) and received using an R&S HF907 (Rohde & Schwarz GmbH & Co. KG, Munich, Germany) horn antenna (impedance 50 Ω, max. input 300 W).

The signal strength was measured and recorded using an Agilent N9038A MXE EMI receiver (Agilent Technologies, Santa Clara, CA, USA). The distance between the transmitting and receiving antennas was set to 2 m. Each measurement was repeated three times, and the values were averaged to reduce experimental error. The shielding effectiveness (SE), reflection (R), and absorption (A) were calculated according to the IEEE standard method for measuring shielding effectiveness [26]. Absorption was determined by calculation.

**Figure 1:**
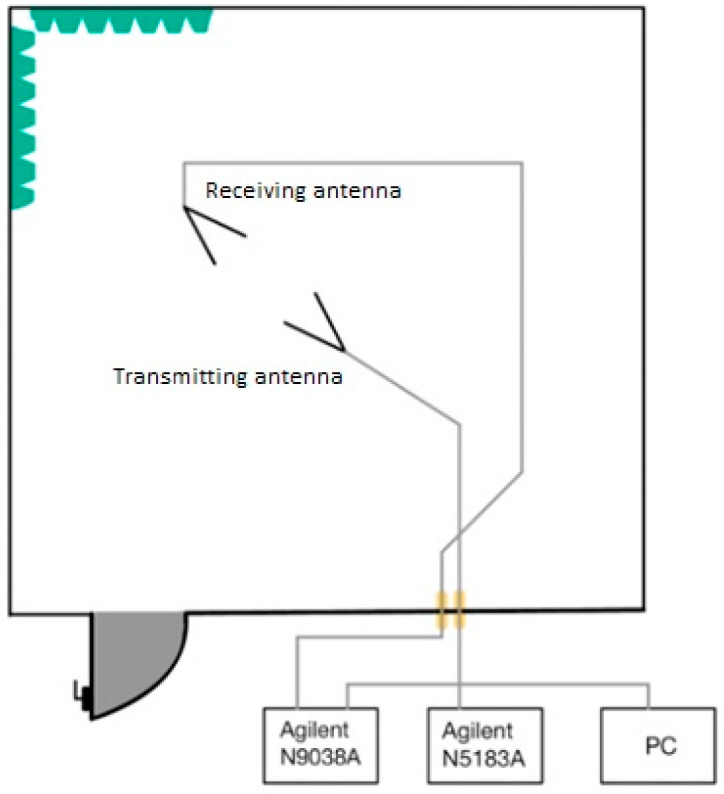
Calibration of the workplace.

To achieve the best measurement results, it was necessary to properly calibrate the workplace. Two antennas, an Agilent N9038A MXE EMI (Agilent Technologies, Santa Clara, CA, USA) spectrum analyzer, and an Agilent N5181A (Agilent Technologies, Santa Clara, CA, USA) pulse generator were calibrated. The connection was made according to the block diagram (Figure 1) [25,26].

The entire calibration was based on the radio communication equation, which can be written as

*P*_p_ = *P*_v_ − *L*_0_ + *G*_v_ + *G*_p_ (4)

*P_P_* is presented as the received power, *P*_V_ is the power transmitted, *L*_0_ is the loss in free space, *G*_P_ is the gain of the receiving antenna, and *G*_v_ is the gain of the transmitting antenna. The loss occurring in free space can be calculated as

*L*_0_ = 20*log*((4*πR*)/*λ*) (5)

*R* is the antennas’ mutual distance, and *λ* is the wavelength. The wavelength can be calculated using the formula

*λ* = *c*/*f* (6)

where *f* is the wave’s frequency and *c* is its propagation speed in vacuum. In the calculations, it is also necessary to consider the attenuations that occur in the cables used for measurement. Therefore, it is necessary to consider additional losses. Ideally, the formula above for *L*_0_ is sufficient. In practice, the total losses in space are calculated as

*L* = *L*_0_ + *L*_A_ (7)

*L*_0_ represents the losses incurred in free space, and *L*_A_ is presented as additional losses. Additional losses consist of several components, which apply to the transmission between two antennas. It can be expressed as

*L*_A_ = *L*_FTx_ + *A*_AG_ + *A*_RAIN_ + *L*_POL_ + *L*_POINT_ + *L*_FRx_ (8)

*L*_FTx_—losses between the transmitting antenna and the transmitter’s output (switches, conductor attenuation).
*A*_AG_—attenuation caused by the ionosphere and atmosphere.
*A_RAIN_*—attenuation caused by clouds and rain.
*L*_POL_—losses caused by the polarization change between the receiving and transmitting antennas.
*L*_POINT_—losses arising from inaccurate antenna-pointing at the satellite.
*L*_FRx_—losses introduced between the receiver input and the receiving antenna. In the case of measurements in an anechoic chamber, *A*_AG_, *A*_RAIN_, and *L*_POINT_ can be neglected. The essence of the calibration consisted of the correct rotation, placement, and adjustment of the antenna heights so that the equality of the relationship for *P*_P_ applies.

## 5. Results of the Experiments

The measurement was performed in an anechoic chamber, COMTEST Model 1710-100 (COMTEST Engineering, Zoeterwoude, The Netherlands). The Agilent N5183 Pulse Generator (Agilent Technologies, Santa Clara, CA, USA) was used to generate the signal. The signal processing was performed using the Agilent N9038A MXE EMI (Agilent Technologies, Santa Clara, CA, USA). The R&S HF907 (Rohde & Schwarz GmbH & Co. KG, Munich, Germany) antenna was used to receive the signal, and the RF Spin DRH18-E antenna was used to transmit the signal. Each measurement was repeated three times, and their values were averaged. The distance between the individual antennas was 2 m. Measurements were performed on each sample from both the front and back sides. The room temperature during the measurement was 23.2 °C, and the relative humidity was 39.3%. Before the measurement, it was necessary to measure the background reference value to subsequently calculate the attenuation of electromagnetic radiation. This reference value was measured three times, and the resulting values were averaged. The amplitude was set to 17.88 dBm. The underlying measurement data in xlsx. format are available in the Supplementary Materials.

### 5.1. Measurement Results—Shielding Effectiveness

**Figure 2:**
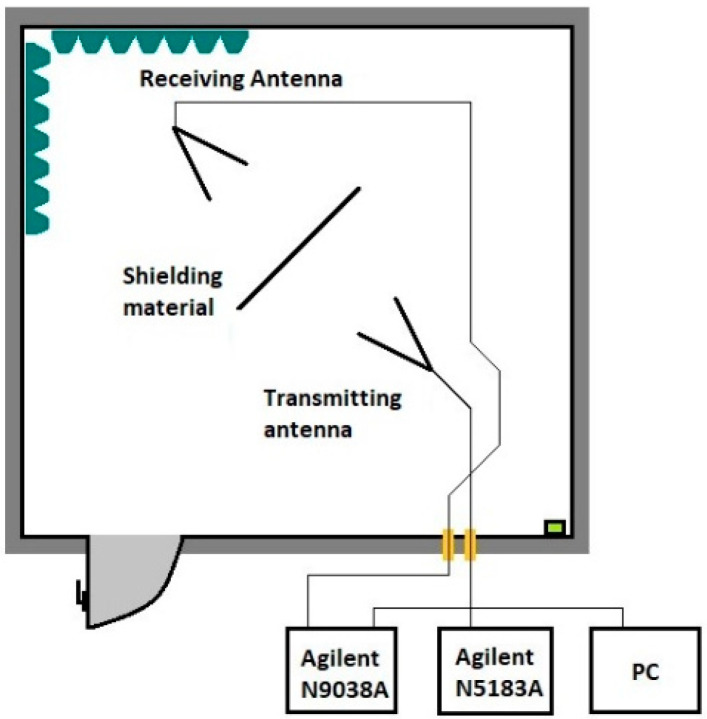
Experiment setup for measuring the shielding effectiveness.

Figure 2 shows the arrangement of the antennas in the anechoic chamber for measuring the shielding effectiveness.

**Figure 3:**
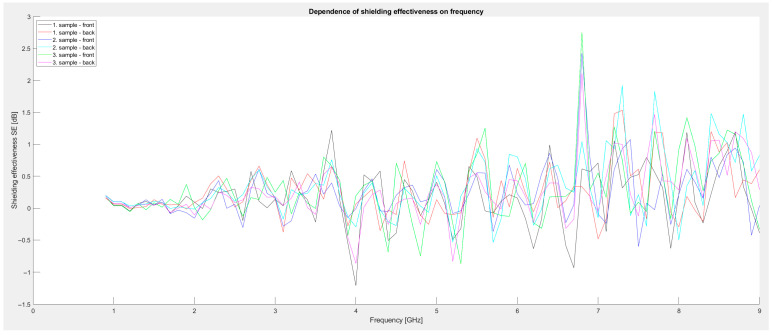
Graphical dependence of shielding effectiveness on frequency (first series of measurements).

First series of measurements: A total of 400 mL of Primalex Plus paint and three metal powders weighing 30 g were used. Manganese dioxide, iron, and aluminum oxide powders were mixed into individual measuring cylinders. The graph in Figure 3 shows their shielding effectiveness. For the first sample, MnO_2_ powder was used, which is manganese dioxide. The second sample was created from Primalex Plus paint and Fe powder. The Fe powder was enriched with silicon to prevent corrosion of the metal and air humidity or water itself, which is also contained in Primalex Plus. The third sample was created by combining Primalex Plus and 30 g of aluminum oxide Al_2_O_3_ powder. The orientation of the plate during the measurements was such that in one case, the coating was oriented opposite the transmitting antenna (sample-front) and in the second case, opposite the receiving antenna (sample-back). As can be seen from the graph, the shielding levels in this series are low, ranging mainly between −1 and +1 dB. In general, there were no significant differences between the front and back sides of the samples, indicating a relatively homogeneous application of the coating. A slight increase in shielding was observed around 7 GHz, especially in the sample containing iron particles, which may be related to their magnetic properties and suitable dispersion in the water-based paint.

**Figure 4:**
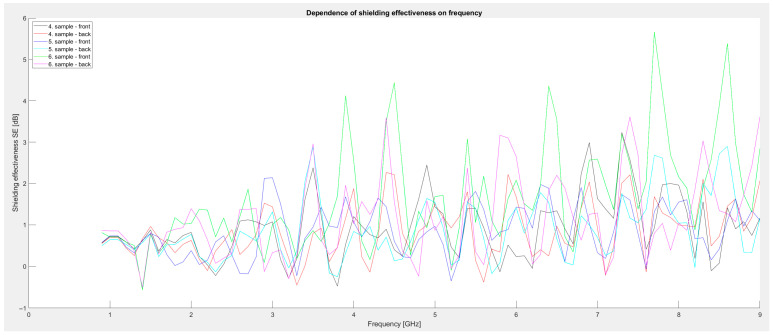
Graphical dependence of shielding effectiveness on frequency (second series of measurements).

Second series of measurements: Metal powder composed of iron powder, aluminum oxide, and manganese dioxide powder was used. The amount of additives was 50 g vs. 400 mL of paint (sample 4). The next sample was 75 g each of Fe powder and zinc oxide (sample 5). The third sample contained 100 g of powdered graphite, 250 g of Fe powder, 250 g of aluminum oxide, and 100 g of manganese dioxide (sample 6). The graph in Figure 4 shows a comparison of the individual samples. Compared to the first series, these samples show significantly higher shielding values, reaching local maxima of up to 5.6 dB, especially in the 3.5–8.6 GHz range. Sample 6 shows improved results (especially in the front-side measurement), which showed the highest SE values at several frequencies above 6 GHz. Sample 5 also demonstrated comparable performance in certain frequency regions. These results indicate an improvement compared to the first series, likely due to the higher concentration of conductive and magnetic components in the coating composition.

**Figure 5:**
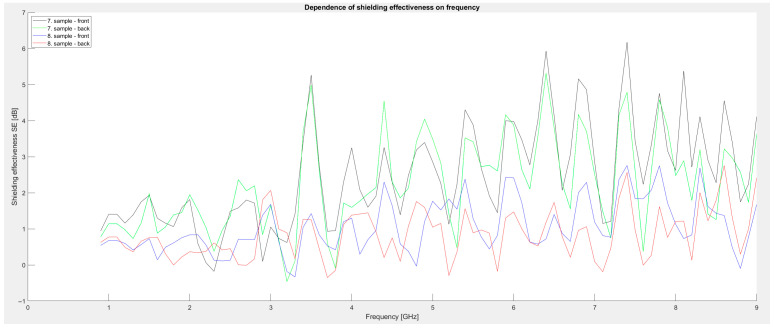
Graphical dependence of shielding effectiveness on frequency (third series of measurements).

Third series of measurements: Powdered graphite was added to the metal powders. For the seventh sample, 100 g of powdered graphite, 100 g of iron powder, and 100 g of manganese dioxide were used. The last eighth sample consisted of 75 g of graphite and 75 g of aluminum oxide added to 400 mL of Primalex Plus paint. A comparison of the individual samples is shown in the graph in Figure 5. The graph clearly shows that the third series achieves the highest shielding values among all series. Shielding exceeds 3 dB over a wide frequency range, and in sample 7, local values of up to 6.2 dB are achieved, specifically in the frequency around 7.4 GHz. These results confirm that the presence of graphite as a conductive filler significantly contributes to increased electromagnetic absorption and also acts synergistically with iron particles and metal oxides. The increase in efficiency at higher frequencies is likely related to the increased conductivity of the coating and its ability to absorb high-frequency components of EM radiation. Minimal differences between the front and back sides indicate homogeneity of application.

Based on these results, it can be concluded that the addition of powdered graphite is an effective strategy for increasing the effectiveness of electromagnetic shielding, with sample No. 7 achieving the best results of all the tested compositions.

### 5.2. Measurement Results—The Reflection of the Electromagnetic Field

The measurement of the reflection of electromagnetic radiation was carried out similarly to the measurement of the shielding effectiveness in the anechoic chamber, but with a different antenna arrangement. The antennas were placed in front of the measured sample and at a certain angle to each other. The antennas were approximately 100 cm away from the samples and were at the same height as in the previous measurement. The amplitude value remained the same as in the measurement of the shielding effectiveness at 17.88 dBm. A similar formula can be used to calculate the reflection and shielding effectiveness.

**Figure 6:**
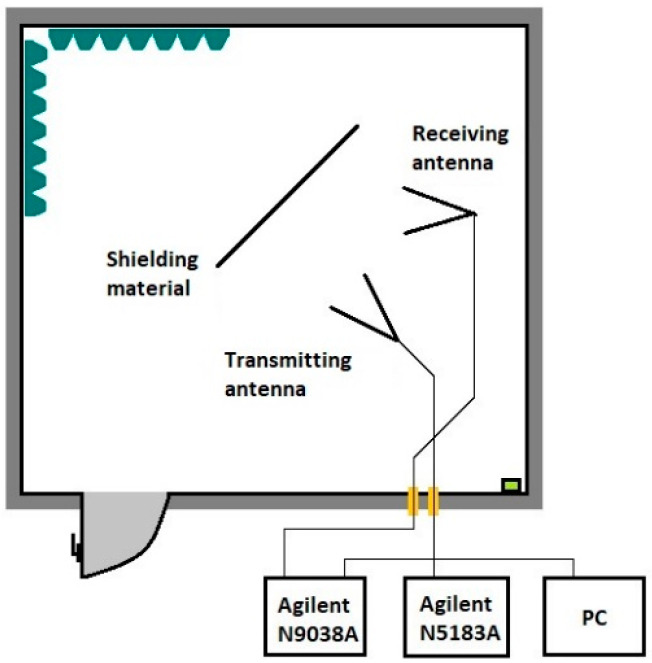
Experiment setup for measuring the reflection of the electromagnetic field.

*R* = |*E*_1_| − |*E*_2_| [dB] (9)

where |*E*_1_| is the electromagnetic wave reflected from the material without shielding and |*E*_2_| is the electromagnetic wave reflected from the material with shielding coating. Figure 6 shows the arrangement of the antennas in the anechoic chamber. The frequency range was the same when measuring the shielding effectiveness from 0.9 GHz to 9 GHz with a step of 0.1 GHz.

**Figure 7:**
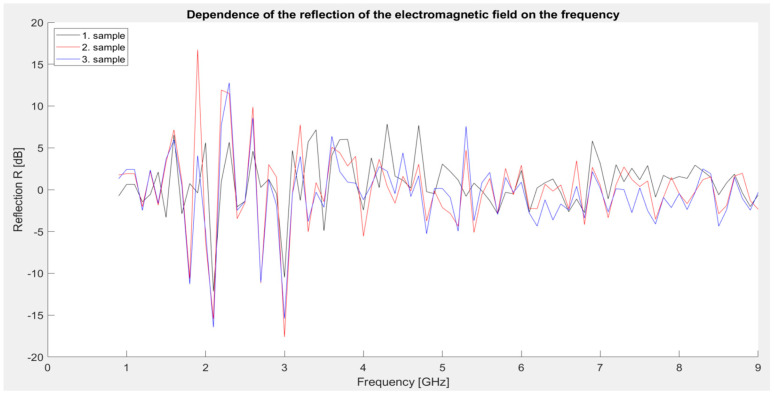
Graphical dependence of the reflection of the electromagnetic field (first series of measurements).

First series of measurements: Identical samples 1, 2, and 3 were used to measure the shielding effectiveness. The samples consisted of Primalex Plus interior paint and 30 g of various metal powders—specifically MnO_2_, Fe, and Al_2_O_3_. The following graphical dependence (Figure 7) represents the reflection values of the mentioned materials.

The most significant reflection values occur in the 2–4 GHz band, where individual samples reach a local maximum of up to ~17 dB (sample 2). Reflection values in higher frequency ranges (5–9 GHz) are significantly lower and oscillate between 0 and 5 dB. All three samples show a very similar trend with slight differences—the highest reflection was observed in sample No. 2, which corresponds to its higher conductivity and ability to reflect incident radiation.

The results suggest that the reflection mechanism dominates over absorption in this series, which corresponds to the low concentration of conductive/magnetic fillers. Higher reflection in these samples means that electromagnetic energy is reflected rather than absorbed, which affects the overall shielding effectiveness—although blocking is increased, so is the propagation of radiation into the surrounding area.

Second series of measurements: Samples identical to those used for measuring shielding effectiveness (4, 5, and 6 samples).

**Figure 8:**
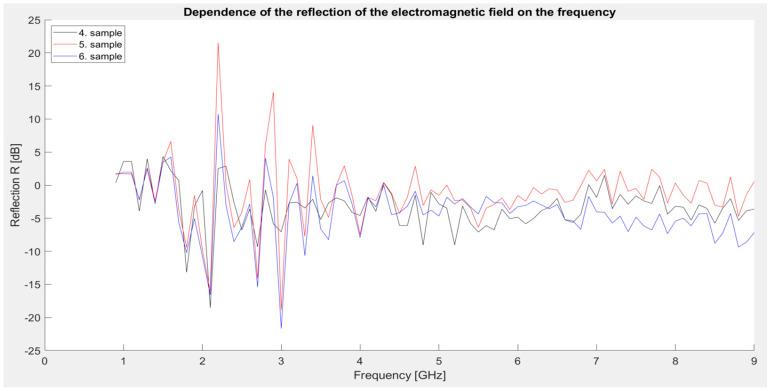
Graphical dependence of the reflection of the electromagnetic field (second series of measurements).

The graph (Figure 8) shows that all three samples exhibit significant dynamics in the 1.5–4 GHz range, with the most pronounced range being between 2.1 and 2.2 GHz. At 2.1 GHz, there is a sharp drop to negative reflection values (down to −16 dB, 5 sample), indicating significant absorption or phase shift of EM radiation in this range. This is immediately followed by a sharp increase in reflection at 2.2 GHz, where sample 5 reaches a maximum of ~22 dB, which is the highest value observed in this series. In the band above 4 GHz, the values are more stable, ranging between −5 dB and +5 dB, with sample 5 again slightly exceeding the others.

These results confirm the observations from the previous shielding measurement—sample 5 shows the highest reflection capacity, probably due to the combination of conductive Fe powder and semiconductive ZnO, which effectively reflect EM radiation in a certain band. A significant transition from negative to positive reflection in the 2.1–2.2 GHz range indicates a resonance effect or a change in the dominant shielding mechanism (from absorption to reflection).

**Figure 9:**
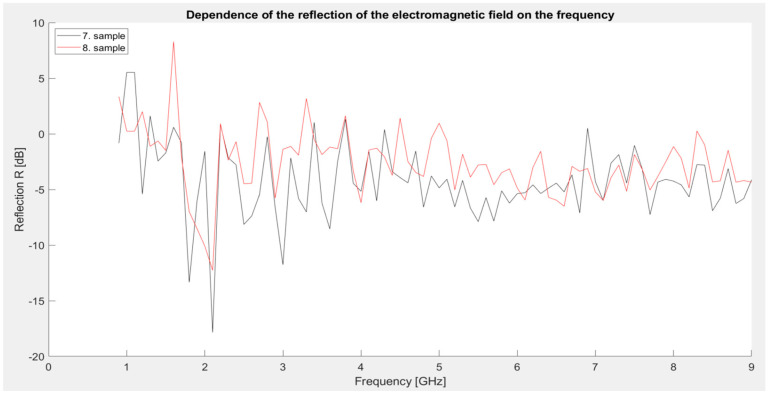
Graphical dependence of the reflection of the electromagnetic field (third series of measurements).

Third series of measurements: Samples identical to those used for measuring shielding effectiveness (third series of measurements). The graph (Figure 9) clearly shows that both samples reach a significant minimum reflection at a frequency of 2.1 GHz, where the values drop to −17.85 dB (sample 7) and −12.25 dB (sample 8), indicating significant absorption in this area. Sample 7 reaches a local maximum in the 1 and 1.1 GHz bands, while sample 8 reaches a maximum at 1.6 GHz. Overall, the reflection values are lower than in previous series, which is positive from the point of view of the application of the coating as an absorption shielding—i.e., one that not only blocks but also attenuates radiation. The difference between samples 7 and 8 is clear—sample 7 shows lower reflection values in a wider frequency range, which corresponds to its better shielding effectiveness observed in previous measurements. This development confirms that a higher iron content together with graphite significantly contributes to the absorption mechanism, while at lower iron concentrations (sample 8) the reflection behavior changes slightly.

### 5.3. Measurement Results—The Absorption of the Electromagnetic Field

The calculation of absorption was made according to the relationship

*A* = *SE* − *R* [dB] (10)

**Figure 10:**
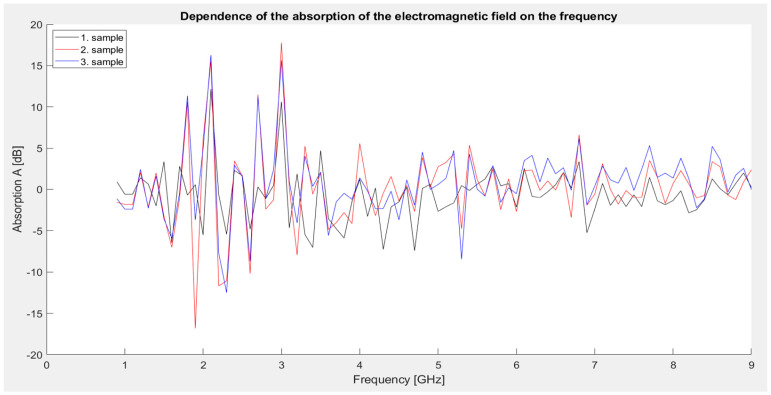
Graphical dependence of the absorption of the electromagnetic field on frequency (first series of measurements).

First series of measurements: Identical samples 1, 2, and 3 as in the shielding effectiveness measurement. The graph (Figure 10) shows that all three samples exhibit a similar absorption pattern, ranging from approximately −16.8 to +17.76 dB. Sample 2 shows the highest fluctuation in values, reaching an extreme minimum of −16.81 dB at 1.9 GHz, which indicates a significant phase shift or interference behavior. In contrast, at 3 GHz, it reaches a maximum of 17.76 dB, which is the highest level of absorption in this series. Sample 3 achieves the most balanced absorption curve across the entire frequency range and provides the best overall results when the values are averaged. Sample 1 (MnO_2_) shows the lowest dynamics of change, and its behavior is relatively consistent, but without significant extremes.

**Figure 11:**
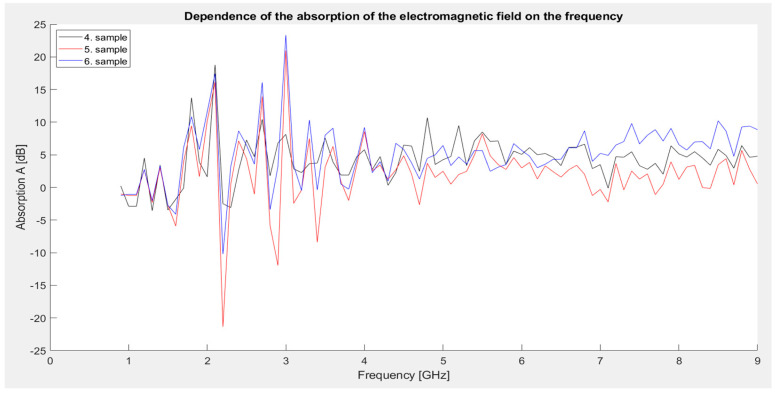
Graphical dependence of the absorption of the electromagnetic field on frequency (second series of measurements).

Second series of measurements:

The graph (Figure 11) shows that sample 6, containing the highest proportion of conductive and magnetic fillers (graphite and iron particles), achieved the highest absorption value in the whole series, up to 23.34 dB, at a frequency of 3 GHz. This result points to a strong absorption mechanism, which is due to a combination of conductivity, magnetic losses, and multiple scattering of electromagnetic energy inside the material. On the contrary, the lowest absorption value was recorded by sample 5 at 2.2 GHz, where the absorption reached up to −21.35 dB, which may be due to resonance or phase interference.

Above 5 GHz, all samples stabilize, with sample 6 continuing to maintain higher average absorption values.

**Figure 12:**
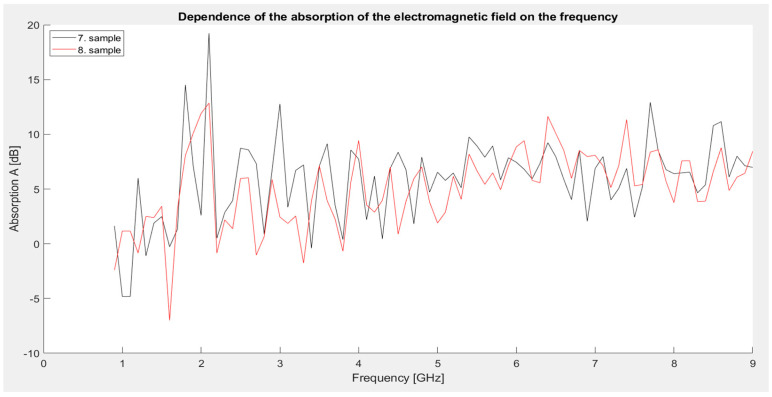
Graphical dependence of the absorption of the electromagnetic field on frequency (third series of measurements).

Third series of measurements:

Looking at the graph (Figure 12), it can be stated that the seventh sample achieved the highest absorption value in this series, namely 19.23 dB at 2.1 GHz, confirming the dominant absorption mechanism at high iron and carbon content. The eighth sample showed the lowest absorption value of −6.99 dB at 1.6 GHz, but at higher frequencies the values were already significantly more stable and positive.

Over the entire frequency range above 3 GHz, both samples achieved relatively balanced values between 5 and 10 dB, indicating their ability to attenuate radiation even beyond resonances.

## 6. Discussion

The aim of this study was to develop a protective coating based on common interior paint with the addition of commercially produced and recycled metal powders and carbon with practical application. The powder mixtures were chosen to represent combinations of various electromagnetically active and passive components. The aim was to identify the most suitable composition in terms of shielding effectiveness based on randomly designed combinations. Fe powder and manganese dioxide (MnO_2_) were used as magnetically active components capable of absorbing electromagnetic radiation. Powdered graphite (C) was chosen as an electrically conductive phase mainly responsible for reflection. Aluminum oxide (Al_2_O_3_) as a non-magnetic and non-conductive component played the role of an insulating filler and at the same time allowed monitoring the influence of non-conductive fillers on shielding. Such an approach allowed for investigating various shielding mechanisms (absorption, reflection, or their combination) and at the same time created a basis for future optimization of the composition of coating mixtures. The best sample (sample 7—100 g C powder, 100 g Fe powder, and 100 g MnO_2_ powder) achieved a shielding effectiveness from 0.38 to 6.2 dB in the full measured frequency range.

In addition to the overall shielding effectiveness, the contributions of absorption and reflection of the electromagnetic field were also analyzed. In the first series of measurements, it was shown that absorption reached a maximum of approximately 17.8 dB at a frequency of around 3 GHz (especially sample 2), while reflection reached its maximum of approximately 16–17 dB at a frequency of 2.2 GHz (sample 2). In the second series of measurements, sample 6 achieved absorption of up to 23 dB at a frequency slightly higher than 3 GHz, which was the best result of this research. Reflection in this research reached a maximum of around 22 dB at a frequency of 2.2 GHz, with sample 5 showing the highest values. In the third series of measurements, sample 7 showed the highest absorption values, namely approximately 19 dB at a frequency of 2.1 GHz, while reflection in this series was lower, generally below 10 dB. Fe powder primarily contributes to absorption through magnetic losses, especially in the lower frequency range up to about 3 GHz. Manganese dioxide (MnO_2_) due to its semiconducting nature and interfacial polarization effects, enhances absorption in the mid- to high-frequency ranges (3–6 GHz). Graphite (C) as a highly conductive material enhances the reflection component throughout the tested frequency range, while alumina (Al_2_O_3_) as a dielectric improves impedance matching and may promote increased absorption by facilitating wave penetration into the material. The combined effect of these fillers results in a broader shielding performance through both reflection and absorption mechanisms.

These results indicate that the tested coatings shield electromagnetic radiation by a combination of absorption and reflection mechanisms, with absorption being the dominant contribution in the better samples. Compared to the literature, where, for example, Farukh et al. [3] report absorption above 30 dB and reflection up to 10 dB for carbon nanotube composites, or Pan et al. [12], where absorption exceeds 80 dB in the X-band, our solution provides lower values but with the advantage of being easy to prepare and for indoor use as a conventional coating in building interiors.

It can also be stated that some coatings showed lower SE (two samples), between 0.5 and 2.5 dB in the entire frequency range, but higher absorption and reflection between 10 and 18 dB, which can be an advantage, for example, when applied in military shelter structures. At the same time, it was shown that it is possible to use recycled materials for such applications, in this case ZnO and Fe. The use of recycled materials improves several environmental and economic impacts. Specifically, reducing the amount of waste in landfilling, reducing the energy intensity by obtaining recycled materials compared to primary processing of raw materials and the associated reduction in financial costs, which will also be reflected in a lower price of the end product.

## 7. Future and Perspectives

Since the possibility of using recycled materials has also been demonstrated, another perspective for the future will be to expand this research to include other mixtures such as Al_2_O_3_, MnO_2_, etc., which will be the output of recycling processes. At the same time, the possibilities of multilayer systems or combinations with other types of materials will be explored, as well as new mixtures of powder materials and their combinations, such as with only carbon filler (C), a mixture of C-Fe-Al_2_O_3_, and others. In the future, it will be necessary to focus on optimizing the composition of the coatings, increasing the concentration of conductive and magnetic components, and testing the mechanical properties, durability, and degradation of the coatings in real conditions.

## 8. Conclusions

This paper aimed to create a protective coating from affordable materials that can be commonly applied in households. Professional protective coatings are available from the company YSHIELD, which has a patent. The problem lies in the price of the product, which is relatively high, but on the other hand, it shows outstanding results in shielding or absorption effectiveness. In our case, a commonly available interior paint from Primalex Plus was used as the main component of the experimental samples, which gradually added various commercially available fabricated metal powders and powders obtained through recycling processes at the Institute of Recycling and Environmental Technologies of the Technical University of Košice. The same grammage of powders was used in the first set of measurements, mixed in 400 mL of Primalex Plus paint. The first series consisted of three samples. The first sample was made from 30 g of manganese dioxide MnO_2_, the second from 30 g of Fe powder, and the third from 30 g of aluminum oxide Al_2_O_3_. In the second series of measurements, the grammage per individual amount of paint was increased, and it also consisted of three experimental samples. The first was created by combining 50 g of iron powder Fe, aluminum oxide Al_2_O_3_, and manganese dioxide MnO_2_. The second sample consisted of 75 g of Fe powder and 75 g of zinc oxide ZnO. The last sample in the second series of measurements consisted of 100 g of powdered graphite, 100 g of aluminum oxide Al_2_O_3_, 250 g of Fe powder, and 100 g of manganese dioxide MnO_2_. Two samples enriched with powdered graphite C were created in the last series of measurements. The first sample in the last series consisted of 100 g of powdered graphite C, 100 g of Fe powder and 100 g of manganese dioxide MnO_2_. The last sample consisted of 100 g of graphite C, 100 g of iron particles Fe, and 100 g of manganese dioxide MnO_2_. Sample 7 achieved the highest shielding effectiveness, mainly due to the synergistic effect of graphite, iron powder, and manganese dioxide. Graphite (C) as a conductive carbon material supports the reflection of electromagnetic waves, while iron (Fe) and manganese dioxide (MnO_2_) contribute to the absorption of radiation through magnetic losses. Their combination ensures a balanced shielding mechanism, where both absorption and reflection are applied. At the same time, this sample maintains a simple and feasible technological process for preparing the coating, which makes it a promising basis for further development. In the future, we plan to investigate in more detail the influence of the ratios of individual components and purposefully adjust the composition to optimize the effectiveness.

## Figures and Tables

**Table 1:** Created protective coatings, composition, and weight of used powders.

	Power Designation	Total Weight
1.	MnO_2_	30 g
2.	Fe	30 g
3.	Al_2_O_3_	30 g
4.	Fe + Al_2_O_3_ + MnO_2_	50 g + 50 g + 50 g
5.	Fe + ZnO	75 g + 75 g
6.	C + Fe + Al_2_O_3_ + MnO_2_	100 g + 250 g + 100 g + 100 g
7.	C + Fe + MnO_2_	100 g + 100 g + 100 g
8.	Fe + Al_2_O_3_	75 g + 75 g

## Data Availability

The original contributions presented in this study are included in the article/Supplementary Materials. Further inquiries can be directed to the corresponding author(s).

## Supplementary Materials

The following supporting information can be downloaded at: https://www.mdpi.com/article/10.3390/ma18163916/s1, Table S1: measurements.xlsx.

## Abbreviations

The following abbreviations are used in this manuscript:

SE	Shielding effectiveness
A	Absorption
R	Reflection
EM	Electromagnetic radiation
dB	Decibel
